# Additive Manufacturing of Cu Using Graphene-Oxide-Treated Powder

**DOI:** 10.3390/ma16155216

**Published:** 2023-07-25

**Authors:** Simon Tidén, Mamoun Taher, Marie Vennström, Ulf Jansson

**Affiliations:** 1 Department of Chemistry—Ångström Laboratory, Uppsala University, Box 538, SE-751 21 Uppsala, Sweden; ulf.jansson@kemi.uu.se; 2 Graphmatech AB, Mältargatan 17, SE-753 18 Uppsala, Sweden

**Keywords:** Cu, graphene oxide, GO, SLM, additive manufacturing, AM, laser powder bed fusion, LPBF

## Abstract

Additive manufacturing of Cu is interesting for many applications where high thermal and electric conductivity are required. A problem with printing of Cu with a laser-based process is the high reflectance of the powder for near-infrared wavelengths making it difficult to print components with a high density. In this study, we have investigated laser bed fusion (L-PBF) of Cu using graphene oxide (GO)-coated powder. The powder particles were coated in a simple wet-chemical process using electrostatic attractions between the GO and the powder surface. The coated powder exhibited a reduced reflectivity, which improved the printability and increased the densities from ~90% for uncoated powder to 99.8% using 0.1 wt% GO and a laser power of 500 W. The coated Cu powders showed a tendency for balling using laser powers below 400 W, and increasing the GO concentration from 0.1 to 0.3 wt.% showed an increase in spattering and reduced density. Graphene-like sheet structures could be observed in the printed parts using scanning electron microscopy (SEM). Carbon-filled inclusions with sizes ranging from 10–200 nm could also be observed in the printed parts using transmission electron microscopy (TEM). The GO treatment yielded parts with higher hardness (75.7 HV) and electrical conductivity (77.8% IACS) compared to the parts printed with reference Cu powder.

## 1. Introduction

Additive manufacturing techniques have shown great potential in rapid prototyping, saving material and manufacturing parts with complex geometries and controlled textures. A material that could be utilized well with complex geometries for heat management systems or electrical components is copper, due to its high electrical and thermal conductivity [1,2,3,4]. The most widely used additive manufacturing (AM) technique for metals is laser powder bed fusion (L-PBF), which uses a laser to melt each powder layer. However, it remains a challenge to print fully dense parts of pure Cu with the most commonly used laser wavelengths in the near-infrared due to the high optical reflectance [5,6,7,8] and thermal conductivity.

A recent review by Jiang et al. [9] has summarized the efforts made in the AM field of pure Cu. Notably, for L-PBF-printed densities above 95%, the power of the laser usually was above 300 W. However, the energy densities for the same printed densities saw a much wider spread between 200–2000 J/mm^3^, indicating that the laser power is the most important parameter, which cannot be truly compensated by smaller layer thickness, hatch spacing, and slower scanning speeds.

Multiple strategies have been employed to facilitate printing of Cu using L-PBF. One of the most common methods is alloying with different elements, which improves processability, but also reduces the desirable high conductivities of pure Cu [10]. High laser powers between 600–1000 W have been used [11,12,13,14,15], but it still remains difficult to print fully dense parts, and lower laser powers yield even lower densities [4,8,16]. Other investigated strategies have been the choice of build plate material [12], powder size distribution [8], and heating of the build plate during processing. Lasers with shorter wavelengths, such as 450 nm blue-diode lasers, have also been used since the reflectance of Cu is much lower in this range [17], but blue high-power continuous wave lasers have only become available in the most recent years and have not been investigated as much.

Other strategies have been to modify the surface of the Cu powders to improve absorption of the laser energy. Jadhav et al. used both a carbon nanoparticle coating [5] and oxidation of the powders [6] separately to reduce the reflectance of the Cu powders, which improved the processability in L-PBF, the former resulting in highest density of ~98% using a 725 W laser power and the latter in ~99% with a 500 W laser. Yang et al. [18] also used an oxidation treatment of the Cu powder surface, which yielded printed parts with ~95% density using only 140 W laser power.

In this paper, we will demonstrate a new concept to print Cu in L-PBF using a powder with reduced reflectivity. This is obtained by a chemical process where the Cu powder is coated with graphene oxide (GO) using differences in pH-dependent surface charges between the functional groups on the GO sheets and the thin surface oxide of the Cu powder [7]. This method promotes full coverage of the powder particle surface instead of agglomerated GO-sheets, which is important for reducing the overall reflectance of the powder. Single-layer graphene has outstanding mechanical properties as well as high electrical and thermal conductivity [19,20]. However, in practice it is difficult to achieve this in a composite material. Additionally, large-scale production graphene is often produced from graphene oxide, which also has good mechanical properties but much lower conductivities. Thermal and electrical conductivity can be improved by reducing the graphene oxide and removing the oxygen-containing functional groups [19].

Graphene–Cu composite powders have been used in conventional sintering, often using ball milling to disperse various forms of graphene in Cu powder before sintering [21], but this does not ensure coverage of all the powder particles and can also lead to changes to the graphene and Cu powder morphology, which is undesirable for powder-bed-based additive manufacturing techniques. Other mixing techniques such as molecular-level mixing, which precipitates Cu particles on the GO-sheets, are both more complicated and also undesirable from a reflectance point of view since the graphene is covered by copper. The sintered graphene–Cu composites can have improved mechanical properties [21,22,23] without reducing electrical conductivity [21] and sometimes even increasing thermal conductivity depending on the alignment of the graphene sheets in the composite [24]. A summary of the material properties of graphene–Cu composites can be found in ref. [21]. Furthermore, Cu forms no carbides with carbon and is actually used as a substrate in graphene manufacturing [25]. Hence, it is possible that L-PBF using a coated powder can give a Cu–graphene composite with new properties.

The aims of this study are two-fold. Firstly, we will demonstrate how GO-treated Cu powder can be used to improve the printability of Cu in L-PBF. The printing process has therefore been studied for varying processing parameters and coating concentrations, and the influence on density and texture has been investigated. Secondly, the changes to the GO during processing has been analyzed.

## 2. Materials and Methods

Pure AM Cu powder (HCCU 0.05% max O, −45 + 15 µm) was gas atomized by Sandvik Osprey and treated by Graphmatech AB with 0.1 and 0.3 wt.% graphene oxide (GO) in separate batches using a wet-chemical process involving pH-dependent electrostatic forces to cover the surface of the Cu powder with GO sheets. The GO treatment process has been described in more detail in a previous publication [7]. The additive manufacturing was performed using an AconityMIDI L-PBF printer equipped with a 500 W single mode fiber laser. Parameters used were 20 µm layer thickness, 300–500 W laser power, 400–1000 m/s scanning speed, 40–120 µm hatch distance, and ~50µm laser spot size. Scanning pattern was a simple stripe, raster pattern rotating 67 degrees every layer. Ar was used as a protective gas and recirculated at 2.2 m/s, and oxygen content was kept below 300 ppm. The highest density for the GO-treated Cu powders was achieved with the processing parameters 500 W laser power, 70 µm hatch distance, and 660 mm/s scanning speed, which was used for printing samples for property measurements. A recoater brush was used to generate the powder layers; no substrate heating was used and both Cu and 316L build plates were investigated, the latter to reduce the heat conducted away through the build plate.

X-ray diffraction (XRD) of powders and polished cross-sections of printed parts was carried out using a D8 Bruker Twin-Twin diffractometer, operated at 40 kV and 40 nA using Cu-Kα radiation. Grinding was performed using SiC paper followed by polishing using 9 µm, 3 µm, 1 µm, and 0.25 µm diamond particle suspensions. Prior to EBSD analysis, the samples were also polished using an OP-S suspension with 2 vol.% H_2_O_2_ and 2 vol.% NH_4_OH.

Powders and microstructures of printed parts were characterized with a Zeiss Merlin scanning electron microscope (SEM) equipped with a Schottky field emission gun (FEG), a Nordlys Max^2^ detector (Oxford Instruments, Oxford, UK), and AZtec HKL software (version 6.0) for electron backscatter diffraction (EBSD) mapping, as well as an X-Max 80 mm^2^ silicon drift detector for energy-dispersive X-ray spectroscopy (EDS). EBSD maps were acquired using 20 kV acceleration voltage and 20 nA beam current with samples mounted on a 70° pre-tilted sample holder.

Characterization of the finer microstructure was performed in a Titan Themis 200 (FEI Company, Eindhoven, The Netherlands) transmission electron microscope (TEM) equipped with probe corrector and four detector SuperX Quantax EDS system (Bruker, Mannheim, Germany). The TEM lamellas were prepared in an FEI Strata DB 235 Focused Ion Beam (FIB) using in situ lift-off. An InVia Raman microscope from Renishaw, using 532 nm laser, was used to analyze the carbonaceous materials both on the surface of the powders and in the printed material. An MMT-X Matsuzawa hardness tester was used to measure the hardness of the printed samples using 100 g load and 13 s dwell time. A SigmaCheck 2 (Ether NDE, St Albans, UK) eddy current conductivity meter was used to measure the electrical conductivity of the printed samples.

## 3. Results and Discussion

**Figure 1:**
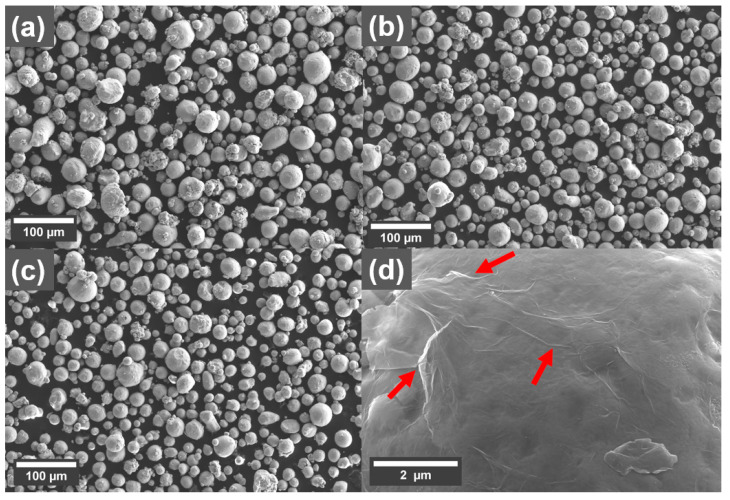
SEM micrographs showing morphology of the Cu powder (**a**) reference, with (**b**) 0.1 wt% and (**c**) 0.3 wt% coating. (**d**) Larger magnification of the surface of the Cu powder treated with 0.1 wt% graphene oxide (GO), with red arrows indicating the presence of wrinkles in the GO sheets.

The morphology of the Cu powder with and without GO treatment is shown as SEM micrographs in Figure 1. There is not an observable change to the size distribution of the powder after the treatment. However, sheet-like structures with wrinkles on them could be observed on the surface of the Cu powders treated with GO sheets (Figure 1d), which is attributed to the presence of GO sheets.

**Figure 2:**
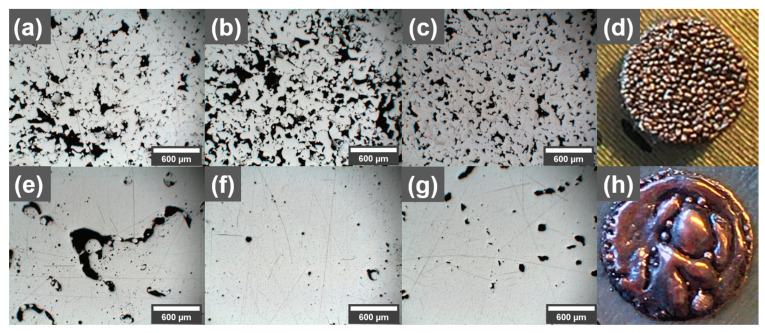
Light optical microscopy images of polished cross-sections (print direction out) of parts printed with (**a**–**c**) reference pure Cu powder and (**e**–**g**) 0.1 wt.% GO-treated Cu powder, using (**a**,**e**) 540 mm/s, (**b**,**f**) 660 mm/s, and (**c**,**g**) 780 mm/s scanning speeds. All parts (**a**–**g**) were printed on 316L build plates using 500 W laser power, 0.07 mm hatch spacing, and 0.02 mm layer thickness. (**d**) Example of balling using low energy input for 0.3 wt.% GO prints. (**h**) Example of over-melting due to too high energy input for 0.3 wt.% GO print.

In an initial printing study with and without GO-treated Cu powder, the printability was investigated using a 316L build plate (see Figure 2). The reference Cu powder showed minimal balling and no spattering, with the main defects of the printed reference parts being lack-of-fusion defects. In general, the prints with Cu reference powder showed a high porosity (Figure 2a–c). In contrast, prints with higher density were obtained with the GO-treated powder (Figure 2d–f). However, the coated powder showed a tendency for balling and spattering (Figure 2d), as well as high surface roughness. The spattering increased with an increase in GO content, which is why the maximum GO concentration was limited to 0.3 wt.% for this study, while the balling was significant at low energy inputs. Since a recoater brush was used instead of a blade, the balling would propagate and grow even larger, further into the print. Prints on the 316L build plate showed a tendency to over-melt in the beginning of the prints, especially for the GO-treated powder using high laser powers above 400 W (example seen in Figure 2h), which could be attributed to alloying between the build plate and the Cu, increasing the energy absorption from the laser. Different process parameters in the beginning of the prints with lower energy input was attempted, but it was difficult to find a suitable tradeoff between over-melting and where balling did not start due the lower energy input after a few layers. The balling behavior could originate from the increased oxygen concentration that the GO brings [26] but could also be a result of carbon enrichment at the surface of the printed part [27,28].

After process optimization, it was found that a stable process could be achieved without too much balling in the process parameters range of 500 W, 500–700 mm/s and 70 µm hatch distance (510–714 J/mm^3^ energy input). Light optical microscopy images of cross-sections from parts printed with reference, 0.1 wt.%, and 0.3 wt.% GO-treated Cu powders are shown in Figure 2. It can be observed that for certain processing parameters, the densities of printed Cu parts can be improved using powders treated with GO because of the reduced reflectance, which allows for more of the laser energy to be absorbed by the GO-treated powder. However, the process appears to be more volatile as the morphology of the reference does not change significantly within the shown process parameter window, but for the printed 0.1 wt.% samples, large differences can be seen. For the higher energy input using 540 mm/s scanning speed, large irregularly shaped pores associated with over-melted regions could be observed. The porosity is decreased as the scanning speed is increased to 660 mm/s, only to be increased again when the scanning speed is increased further to 780 mm/s, possibly to increase spatter, which increased with higher scanning speeds, which was observed for the GO-treated powders.

In an attempt to minimize the effects of over-melting in the beginning of the print, a Cu build plate was also investigated. When printing the reference pure Cu powders, the parts would detach from the Cu build plate due to insufficient melting of the first layers. However, both the 0.1 and 0.3 wt.% GO-treated Cu powders were both processable on the Cu build plate. In general, higher density was observed using a Cu build plate. The 0.1 wt.% GO Cu samples had lower porosity than the 0.3 wt.% GO Cu samples, likely due to the increased spatter observed for 0.3 wt.% GO concentration. This indicates that there is a trade-off in the GO concentration, with too high concentration resulting in process instabilities due to spatter.

**Figure 3:**
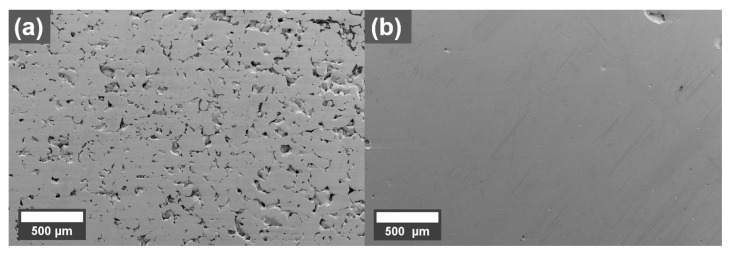
SEM micrographs of cross-sections of printed (**a**) reference Cu printed on 316L build plate and (**b**) 0.1 wt.% graphene oxide treated Cu printed on Cu build plate, (build direction out).

SEM micrographs of cross-sections perpendicular to the build direction of parts printed with the best parameters for the 0.1 wt.% coated Cu powder is shown in Figure 3. The printing parameters, selected inside the optimized process window, were 500 W laser power, 660 mm/s scanning speed, and 0.07 mm hatch distance. The coated powder was printed on a Cu build plate (Figure 3b) but since it was impossible to obtain good builds on this plate with untreated powder, a 316L plate was used for the reference Cu sample (Figure 3a). The results in Figure 3 show that the 0.1 wt.% coating resulted in a denser part compared to an uncoated powder. The density of the 0.1 wt.% sample is estimated to 99.8% using image analysis while the reference shows clear lack of fusion defects and a density of 89.0%.

**Figure 4:**
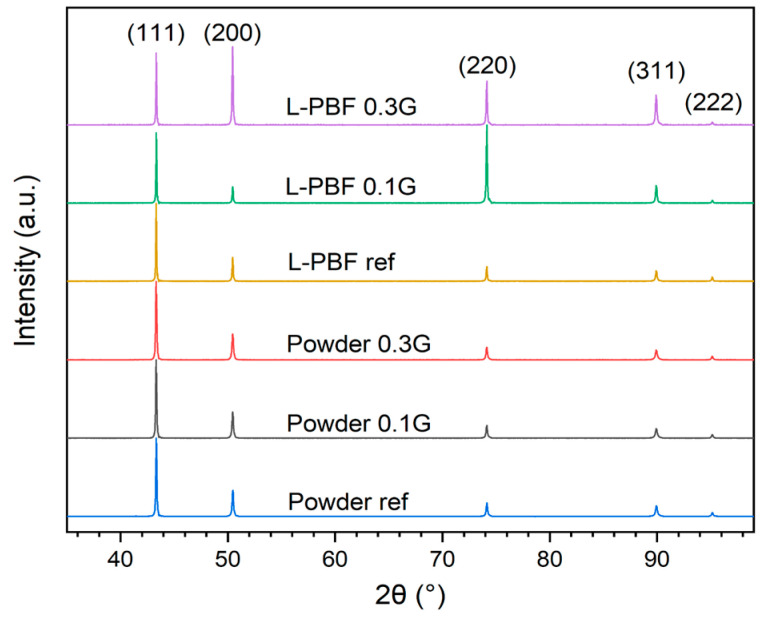
XRD of the Cu reference, 0.1 wt.%, and 0.3 wt.% treated powders and L-PBF printed parts.

X-ray diffractograms of the reference and GO-treated powders, as well as their respective printed parts, are shown in Figure 4. The diffractograms for the powders are all similar even after the GO treatment, without peaks that could be attributed to oxides or agglomerated carbon sheets. However, for the diffractograms of the polished printed parts with scattering vector parallel to the build direction, a difference in texture could be observed for varying GO concentration. The diffractogram of the printed reference is very similar to the powders, attributed to the incomplete melting, fast cooling rate, and therefore random distribution of the grains. The 0.1 wt.% sample showed a higher intensity of the (220) peak, indicating a stronger <110> texture along the build direction, while for the 0.3 wt.% sample both the (200) and (220) peak have relatively higher intensity than for the powders and the printed reference. This indicates a stronger texture in both <100> and <110> along the build direction.

**Figure 5:**
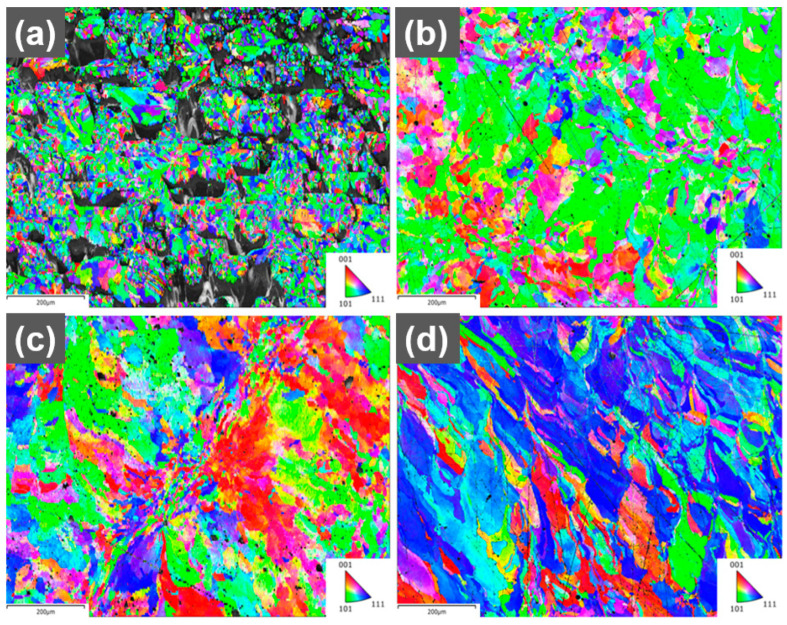
EBSD maps of grain orientation for printed (**a**) reference (build direction out), (**b**) 0.1 wt.% (build direction out), (**c**) 0.3 wt.% (build direction out), (**d**) 0.1 wt.% (build direction up), scale bars 200 µm.

A more detailed analysis of the texture in the printed parts was performed using EBSD (Figure 5). The grains in the reference part are much smaller, which is attributed to the incomplete melting and quick cooling of the material. The 0.1 wt.% part shows many grains with <110> orientation along the build direction, which is consistent with the results of the X-ray diffraction. The printed 0.3 wt.% shows fewer grains with <110> orientation along the build direction compared to the 0.1 wt.% sample. The difference in textures for 0.1 wt.% and 0.3 wt.% could be explained by different laser absorption leading to a different shape of the melt pool and a different temperature gradient. A deeper melt pool due to higher absorption from the increased GO concentration could increase the amount of <100> grains and reduce the amount of <110> grains along the build direction for face-centered cubic (FFC) materials [29].

**Figure 6:**
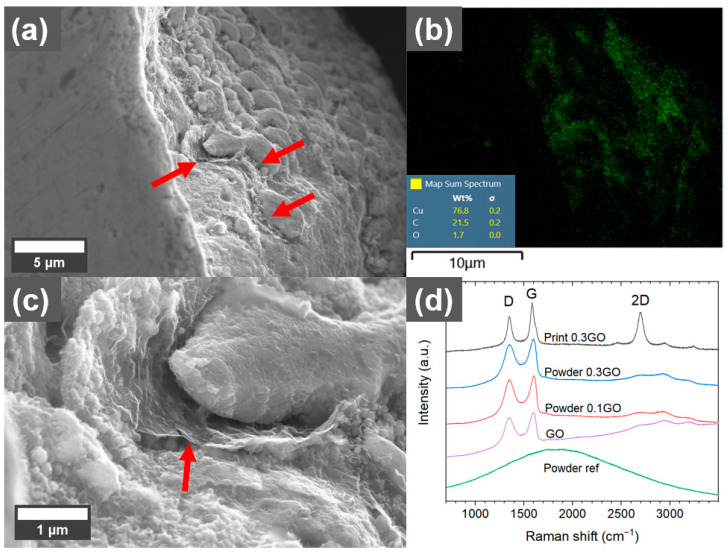
(**a**) SEM micrograph of sheet-like structure (indicated with red arrows) in the pore of a part printed with 0.3 wt.% graphene oxide (GO)-treated Cu powder using 500 W laser power. (**b**) EDS mapping of the area shown in (**a**) with green color indicating the carbon-rich areas. (**c**) Higher magnification of the sheet-like structure. (**d**) Raman spectra of reference, 0.1 wt.%, and 0.3 wt.% GO-treated powders and the sheet-like structures in pores of the printed material using 500 W laser power.

Cu has a very low solubility of carbon and form no carbides. In some areas of the cross-sections of the printed coated Cu, such as in pores, sheet-like structures could be observed (Figure 6). EDS analysis showed that it was mainly carbon, indicating that some of the GO sheets remained in the printed parts after processing in L-PBF. Raman analysis of other pores (Figure 6d) showed changes in the Raman spectrum compared to the GO before printing. Both the D band (~1340 cm^−1^), attributed to breathing modes of six-atom rings, which require defects to activate, and the G band (~1595 cm^−1^), attributed to the bond stretching of sp^2^ bonds, are narrower after printing [30]. Additionally, a reduced D/G intensity ratio and increased intensity of the double resonance 2D band (~2690 cm^−1^) could be observed after printing (compared with the reference spectrum of the as-received GO). The 2D band at ~2690 cm^−1^ is typical for graphene and graphite and indicates that a reduction of the GO sheets occurs during processing in L-PBF [31], which is in line with the high carbon content compared to the oxygen content in the EDS mapping of the area.

**Figure 7:**
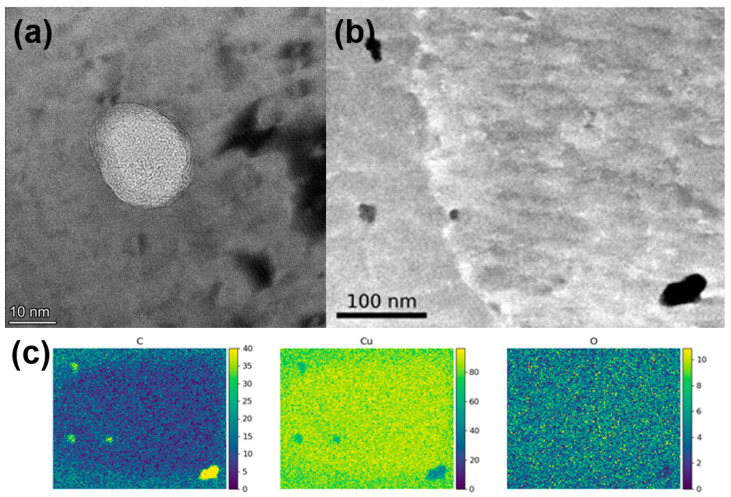
(**a**) TEM bright field micrograph showing inclusions inside the Cu matrix filled with layered material. (**b**) STEM micrograph showing four inclusion and one grain boundary. (**c**) EDS mapping (at%) of the area in (**b**), showing carbon enrichment in the inclusions.

TEM was used to further investigate the morphology of the remaining carbonaceous material inside the printed parts, which is shown in Figure 7. Small inclusions ranging between ten nanometers to several hundred nanometers in diameter could be observed. Layered crumpled structures could be observed within these inclusions, which were confirmed to be carbon using EDS mapping. These small carbon-filled inclusions appeared to be randomly distributed and did not seem to appear more often in grain boundaries.

**Figure 8:**
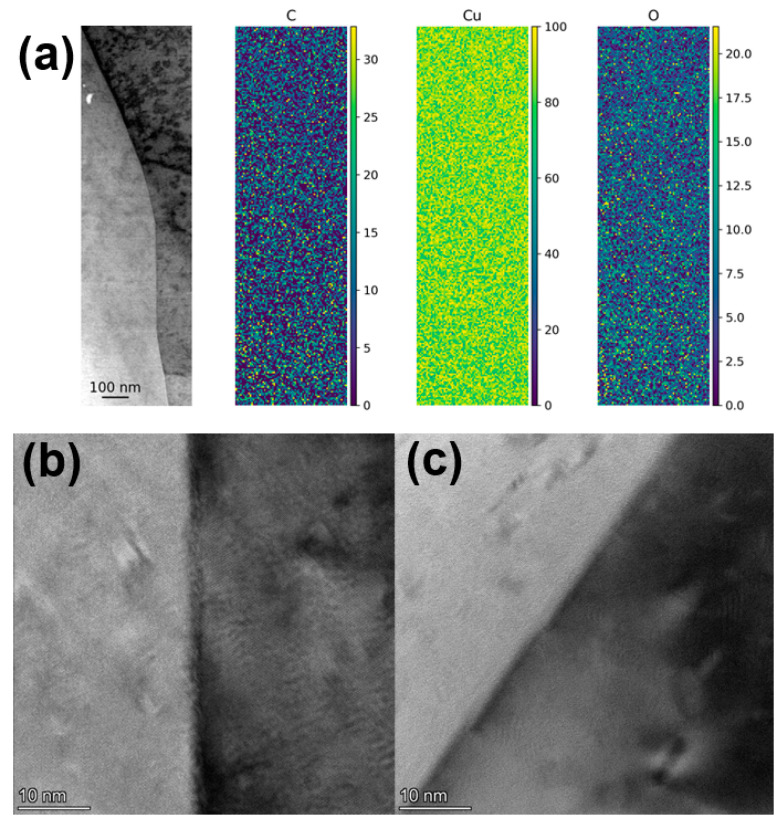
(**a**) STEM micrographs with EDS mapping (at%) and (**b**,**c**) HR-TEM micrographs of grain boundaries in parts printed with 0.3 wt.% graphene oxide-treated Cu powder at 500 W laser power.

One possibility during melting in the L-PBF process is that some of the carbon gets dissolved in the Cu as it is heated up and melted. The solubility of C in Cu is extremely low at room temperature but increases as the Cu is heated [32]. When melted Cu cools down and solidifies, the dissolved C must then be pushed out of the Cu phase. In fact, Cu is used as a substrate to grow graphene from a carbon-containing atmosphere at high temperatures (see, e.g., ref. [33]). Therefore, one could assume that there would be an enrichment of C and potentially graphene formation in the grain boundaries giving a Cu/graphene composite after the cooling down process. Multiple grain boundaries were therefore examined with HR-TEM and STEM EDS mapping, shown in Figure 8. However, no layering or carbon-enrichment could be detected in the grain boundaries that were examined.

**Figure 9:**
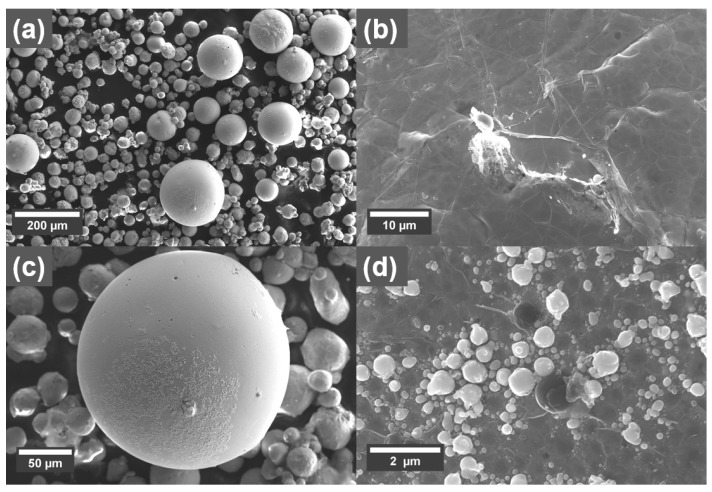
SEM micrographs from a 0.3 wt.% coated Cu powder showing (**a**) spatter particles. (**b**) Sheet-like structures on the surface of a larger spatter particle. (**c**) One large spatter particle with small sub-micrometer particles on its surface. (**d**) High magnification of the smaller sub-micrometer particles on the surface of a larger spatter particle.

As described above, spattering could be observed with the coated powder. SEM micrographs of spatter particles produced from 0.3 wt.% GO-treated Cu powder processed with 500 W laser are shown in Figure 9. The spatter particles are of varying size with the largest being around 200 µm in diameter (Figure 9a). Interestingly, on most of the surface of the spatter particles, sheet-like structures could be observed, indicating the presence of graphene-like sheets (Figure 9b). Furthermore, smaller spatter particles in the sub-micrometer range could also be observed on some areas on the larger spattered particles (Figure 9c,d).

**Figure 10:**
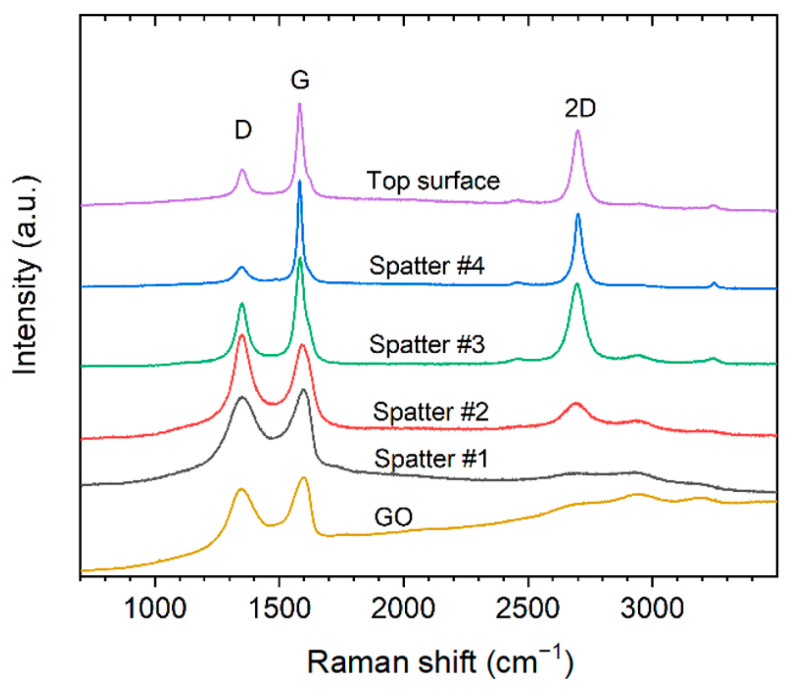
Raman spectra of as-received graphene oxide, different spots on spatter particles (0.3 wt.% GO-treated Cu powder and 500 W), and top surface of printed part processed at 500 W laser power using 0.3 wt.% GO-treated powder. Showing the typical D (~1350 cm^−1^) and G (~1600 cm^−1^) bands and overtone bands in the 2400–3200 cm^−1^ region of graphene-like materials.

Raman analysis of the surface of the spatter particles confirm that they are covered with graphene-like sheets (Figure 10). The Raman spectra showed a large variance, as some regions had significantly stronger 2D and G bands with lower intensity D bands, while other regions showed Raman spectra more similar to that of the GO and the GO-treated Cu powders before printing (Figure 6d). A selected example of Raman spectra from different regions of the spatter particle surfaces are shown in Figure 10. Additionally, the Raman spectra from the top surface of the printed parts is added together with a reference spectrum of GO used for the treatment. The results indicate that the spatter particles are covered with graphene-like sheets with varying degree of reduction from GO to a more reduced state.

The carbon and oxygen content for L-PBF printed parts from the reference, 0.1 wt.% and 0.3 wt.% powders are shown in Table 1. The oxygen content in the printed parts decreases with increasing amount of GO on the Cu powders. This could be attributed the carbon in the GO acting as a reducing agent for the Cu oxide. The higher porosity for the Cu reference could also be a reason for its higher oxygen content, due to more internal interconnected pores that have oxidized after the parts have been taken out of the print chamber. It is also evident from the carbon content that most of the GO is not remaining inside the printed parts, either due to floating to the top of the melt pool or due to being evaporated due to the high laser power. The 0.1 wt.% GO sample showed similar carbon content compared to the reference Cu, but increased threefold to 0.018 at.% C for the 0.3 wt.% sample.

**Figure 11:**
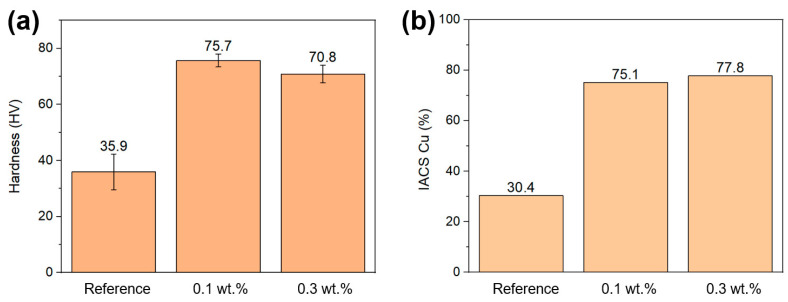
(**a**) Hardness (Vickers) measurement of reference, 0.1, and 0.3 wt.% printed Cu samples. (**b**) Electrical conductivity of printed reference, 0.1 and 0.3 wt.% GO-treated Cu samples, compared to the IACS.

Hardness measurements on printed parts are shown in Figure 11. The low hardness of the printed reference (35.9 HV) can be attributed to the high porosity of this sample. The 0.1 wt.% printed sample with 99.8% density showed a hardness of 75.7 HV, which is harder than previously reported for L-PBF printed pure Cu [5,6], but lower than the oxide-dispersed strengthened L-PBF Cu at 91 HV [6]. Furthermore, it is harder than previously reported to process pure Cu in electron beam-PBF (EB-PBF) due to the increased processing temperature that leads to a coarser microstructure in EB-LPBF. [2] The 0.3 wt.% sample shows a decrease in hardness to 70.8 HV compared to the 0.1 wt.% sample, which is likely attributed to the higher porosity. The electrical conductivity of the reference was 30.4% of the International Annealed Copper Standard (IACS, 100% being 58 MS/m), which was increased to 75.1 and 77.8% for the 0.1 wt.% and 0.3 wt.% samples, respectively. The observed increase is mainly attributed to the increase in density and larger grain size. Not reaching 100% of IACS conductivity for the relatively dense 0.1 and 0.3 wt.% samples could be due to the grain size of the printed parts, and annealing of these samples could increase the electrical conductivity.

## 4. Conclusions

In this study, we have used a fast and cost-effective process to coat Cu powder with graphene oxide (GO) using electrostatic interactions between the metal surface and the GO sheets. The GO treatment improved the printability of the Cu powder due to the increased energy absorption from the laser. Due to this, it was possible to increase the density of printed Cu compared to uncoated powder. Samples with 99.8% density were printed with 0.1 wt.% GO treated powders after optimization of laser power, scanning speed, and hatch distance. The printability of the GO-treated Cu powder did not increase with increasing GO concentration, with 0.1 wt.% resulting in denser samples and less spatter particles compared to 0.3 wt.%. The GO-treated Cu powders also showed a tendency to over-melt at high laser powers especially in the beginning of the prints on a 316L build plate. Various carbonaceous materials could be found inside the printed material, both sheet-like structures in the same size-range as the original GO, which indicates the survival of some graphene-like material and carbon-enriched inclusions in the size range of 10 nm to several hundred nm. The improved densification led to increased hardness and electrical conductivity for GO-treated samples, but it was not possible to separate the density and GO sheet contributions.

The coating process utilized in this study is easily applied to a low cost and on a larger scale. This suggests that the approach can be used for an industrial production of printed Cu components with L-PBF.

## Figures and Tables

**Table 1:** Carbon and oxygen content in atomic percent (at.%) for L-PBF parts printed with reference, 0.1 wt.%, and 0.3 wt.% Cu powders.

Sample	Carbon (at.%)	Oxygen (at.%)
Reference Cu	0.005	0.075
0.1 wt.%	0.005	0.017
0.3 wt.%	0.018	0.010

## Data Availability

Data will be made available on request.
